# Pheochromocytoma in an accessory adrenal gland: a case report

**DOI:** 10.4076/1757-1626-2-6271

**Published:** 2009-08-03

**Authors:** Oludolapo Afuwape, Josephus K Ladipo, Olabiyi Ogun, Jokotade Adeleye, David Irabor

**Affiliations:** 1 Department of Surgery, University College HospitalIbadanNigeria; 2 Department of Pathology, University College HospitalIbadanNigeria; 3 Department of Medicine, University College HospitalIbadanNigeria

## Abstract

Very few cases of pheochromocytoma in functional accessory adrenal glands have been documented in literature. We present a twenty-four year old Nigerian female who presented with pheochromocytoma. Investigations revealed a suprarenal mass, which was diagnosed as an accessory gland adrenal tumour at surgery. This shows that accessory adrenal glands can be a basis for development of pheochromocytoma.

## Introduction

Pheochromocytomas are catecholamine producing tumours of the chromaffin cells of the adrenal medulla. Similar catecholamine producing tumours of extra-adrenal chromaffin cells are known as paragangliomas. About 85% of pheochromocytomas arise from the adrenal medulla and about 15% arise from extra-adrenal chromafin cells [1]. Only few cases of pheochromocytoma in accessory adrenal glands have been reported in literature. We present a rare case of a patient with pheochromocytoma of an accessory adrenal gland.

## Case presentation

**Figure 1.**
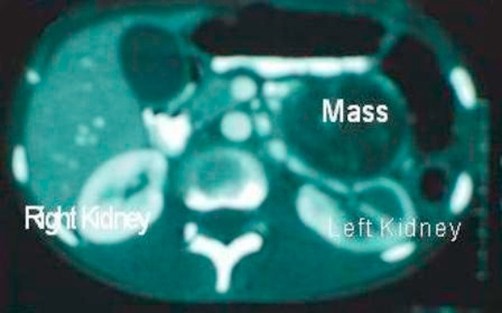
Abdominal CT showing the left kidney and the over lying mass.

A 24-year-old Yoruba Nigerian female presented with a four month history of hypertension and intermittent bilateral frontal headache. There was an associated history of photophobia, tinnitus, palpitation and diaphoresis. She admitted having heat intolerance and insomnia. She had lost weight significantly. There was no history of seizures or loss of consciousness. Examination at presentation revealed an anxious patient. There was no demonstrable anterior neck swelling. Her pulse rate was 100 per minute, regular and the blood pressure was 190/100 mmHg. There were no significant findings in the abdomen. A tentative diagnosis of pheochromocytoma was made. The differential diagnosis was thyrotoxicosis. She was commenced on antihypertensive medications while she was investigated. Results of investigations revealed a normal thyroid function test, an elevated serum cholesterol level (296 mg/dl) and a normal serum urea and electrolytes. An abdominal ultrasound scan revealed a left suprarenal mass displacing the left kidney postero-laterally, suggestive of a left adrenal mass. A computerized tomography scan of the abdomen revealed a rounded bi-lobed left suprarenal mass of mixed density (Figure 1). The twenty-hour urine metanephrine estimations were markedly elevated. A diagnosis of pheochromocytoma was subsequently confirmed. She was commenced on Prazocin 0.5 mg p. o t.d.s and Atenolol 100 mg b.i.d. Nifedipine 20 mg b.i.d was subsequently added. The blood pressure was maintained at 120/80 mmHg. She subsequently had surgery for excision of the tumour using a transperitoneal approach. There was a transient episode of intraoperative hypotension for which she was commenced on dopamine infusion. Findings at surgery were

**Figure 2.**
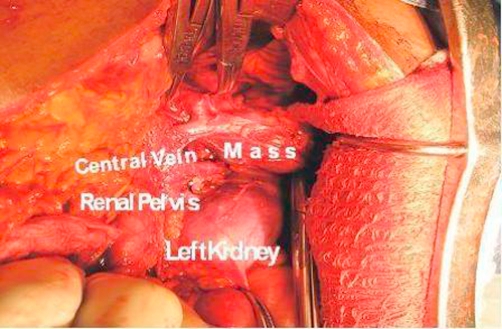
Intra-operative photograph.

- A well encapsulated left suprarenal mass with dilated veins draining into the left renal vein (Figure 2).
- A normal looking left adrenal gland superiorly adjacent to the mass.
- A normal right adrenal gland.

The tumour was dissected out after ligating the blood supply and the veins. A separate cresenteric shaped mass was identified at the apex of the cavity this was also removed.

**Figure 3.**
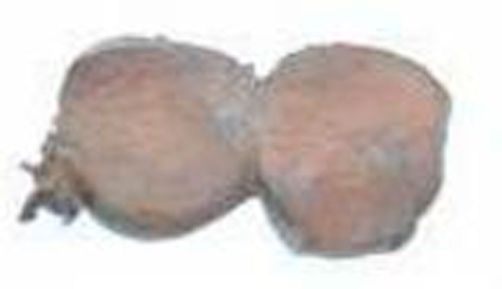
The gross image showing the bi-lobed mass.

**Figure 4.**
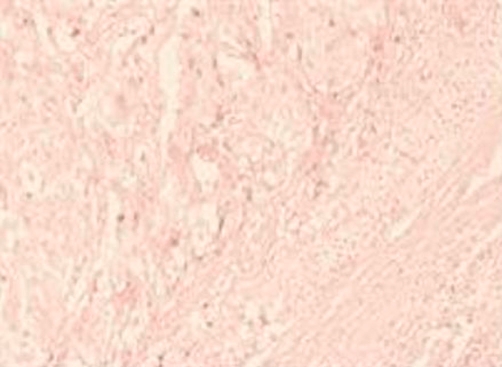
This shows the remnant atrophic adrenal cortical tissue in the right aspect of the photomicrograph and the tumour to the left (×200).

She was transferred to the Intensive care unit for monitoring. Her post-operative phase was uneventful. Anti-hypertensive medications were discontinued and she was discharged home on the ninth post-operative day with a blood pressure of 120/80 mmHg. She is being seen in the surgical out - patient clinic. She has remained free of symptoms. The histopathology report of the two resected specimens revealed a cresenteric shaped normal intact adrenal gland grossly and on histology. The mass (Figure 3) measured 8 × 6 × 4.5 cm in size and weighed 100 gm. The surface was lobulated with sparsely attached adipose tissue on its intact capsular surface. Cut surface revealed a bi lobulated yellowish brown surface. The histology showed a rim of atrophic cortical adrenal tissue and deep to this rim were nests and sheets of small to intermediated sized cells with moderate pleomorphic nuclei with occasional bizarre ones and with moderate amphophillic to eosinophillic cytoplasm (Figure 4). The cells were arranged around delicate vasculature. The chief cells in the tumour were strongly positive for Neuron Specific Enolase, synaptophysin and Chromogranin A. The surrounding sustentacular network of cells stained positively for the S-100 protein. These findings are suggestive of a pheochromocytoma in an accessory adrenal gland.

## Discussion

Accessory adrenal glands are seen in approximately half of newborns but subsequently disappear later [2]. They consist of cortical and glandular tissue and are predominantly in the abdomen and pelvis.

Accessory adrenal glands were initially documented by both Jaffer and Soyer [3]. Graham in a subsequent study demonstrated the incidence of accessory adrenal glands in thirty-two percent of the autopsies done. Sixteen percent of these glands contained both cortex and medulla [4]. The origin of these glands is due to an embryological developmental defect. The accessory adrenal gland separates from the adrenal primodium during migration. The accessory adrenal gland most often consists of cortical tissue. The presence of the medulla is determined by the time of separation from the primodium [3]. Very few cases of pheochromocytoma arising from accessory adrenal glands have been reported. A few reasons attributed to the rarity of pheochromocytoma occurring in accessory adrenal glands are the relatively low occurrence of medullary tissue in these glands; a diminished cellular basis for tumour transformation and the relative inactivity of the medulla in the accessory adrenal glands. In addition the tumour in the accessory adrenal gland tends to cause cortical atrophy when it gets very large consequently causing a misleading diagnosis of paraganglioma [5]. Tumour localization may be initially done with high resolution ultrasonography. Other commonly used imaging techniques include computerized tomography (CT) and Magnetic resonance imaging (MRI) and scintigraphy after the administration of 131 Metaiodobenzylguanidine (MIBG). Although CT may localize about 95% of tumours, MRI is superior to CT in detecting these tumours especially extra-adrenal tumours and metastatic pheochromocytoma. MRI may be considered instead of CT in pregnancy states and in patients allergic to contrast agents. In some centers MIBG is used before CT for tumour localization with recourse to MRI or CT for detailed anatomical studies [6]. Recently, positron emission tomography (PET) with 18Fluorodopamine or 18Fluorodeoxyglucose has been introduced for tumour localization with superior results to MIBG [7]. However abdominal MRI, MIBG and PET are not available in our centre.

The clinical presentation of pheochromocytoma may mimic other disease conditions such as hyperthyroidism and panic disorders consequently causing delay in diagnosis. Biochemical tests remain the hallmark of diagnosis. This includes the determination of urinary metabolites of catecholamine (nor-metanephrine and metanephrine) and urinary vanillylmandelic acid [8]. Preoperative management involves the control of catecholamine induced symptoms with the use of alpha and beta blockers. Calcium channel blockers may also be considered. Intraoperative dangers to be aware of while operating on patients with pheochromocytoma include severe hypotension from a sudden reduction in cathecolamines due to tumour excision and hypoglycemia from rebound hyperinsulinemia. There has been some controversy on ectopic adrenal glands being responsible for clinical manifestation of pheochromocytoma [9]. Our experience with this case supports the fact that pheochromocytoma can occur in an accessory adrenal gland though this is rare. The clinical course and nature of phaechromocytoma in functional accessory adrenal glands are not well documented in literature. However, reports about extra-adrenal paraganglioma have consistently shown that they tend to run a more aggressive course, have a likelihood of metastasis and a high incidence of persistent and recurrent disease [10-14]. Metastasis is the only indicator of malignancy in phaechromocytomas and paragangliomas but the following factors are usually associated with higher incidence of malignancy; tumour weight greater than 80 g, tumour size greater than 5 cm, extra-adrenal location, post operative persistent arterial hypertension, relative young age, male gender and immunohistochemical staining of MIB-1, a proliferative index marker, greater than 3% [11-15].

## Conclusion

This further demonstrates the rare possibility of the development of pheochromocytoma outside the adrenal gland.

## Abbreviations

CT: computerized tomography
MIBG: Metaiodobenzylguanidine
MRI: Magnetic resonance imaging
PET: positron emission tomography
